# Scleroderma Overlapping With Sjögren's Syndrome: A Case Report of a Renal Emergency

**DOI:** 10.7759/cureus.85861

**Published:** 2025-06-12

**Authors:** Suela Mumajesi, Saimir Seferi, Nereida Spahia, Elena Gjecka, Floreta Kurti

**Affiliations:** 1 Nephrology, University Hospital Center "Mother Tereza", Tirana, ALB; 2 Gastroenterology and Hepatology, University Hospital Center "Mother Tereza", Tirana, ALB

**Keywords:** chronic kidney disease, elevated bun. hemodialysis, localized scleroderma, scleroderma renal crises, severe hypertension

## Abstract

Scleroderma renal crisis (SRC) is the most common hallmark of renal involvement in systemic sclerosis (SSc), characterized by acute kidney injury and malignant hypertension. The pathophysiology of SRC involves endothelial dysfunction, activation of the renin-angiotensin-aldosterone system (RAAS), and thrombotic microangiopathy (TMA), leading to renal ischemia and hypertension. When accompanied by overlapping Sjögren's syndrome (SS), the clinical presentation and therapy become considerably more complex. We discuss the diagnosis, management, and outcomes of a patient presenting with SRC and overlapping SS. It highlights the importance of early detection and rapid treatment of SRC, particularly in the presence of overlap syndromes. Early intervention with angiotensin-converting enzyme (ACE) inhibitors and careful monitoring of renal function are crucial for optimal outcomes. The occurrence of overlapping SS complicates the clinical presentation and therapy, emphasizing the importance of an individualized approach to treatment. Healthcare personnel must be especially mindful of this since early detection of renal crisis has a major impact on survival rates and long-term prognosis.

## Introduction

Scleroderma renal crisis (SRC) is a rare but one of the most severe complications of systemic sclerosis (SSc). Its devastating manifestation is characterized by acute kidney injury, malignant hypertension, and thrombotic microangiopathy (TMA). The condition is more usually linked with diffuse cutaneous SSc (10-25%), as opposed to just 1-2% of patients with circumscribed disease, as seen in the case presented in this report [1-4]. SRC typically manifests early in the course of scleroderma, with up to 75% of cases emerging within the first four years after diagnosis [5]. Early diagnosis and aggressive management are crucial to mitigating morbidity and mortality in this patient population [6].

## Case presentation

A 72-year-old Caucasian female presented to the emergency department with severe headaches, nausea, and persistently elevated blood pressure unresponsive to medication. These symptoms had advanced over the past three days and were linked with malnutrition due to difficulty swallowing. Her medical history revealed a diagnosis of limited cutaneous systemic scleroderma (lcSSc) three years prior, for which she was not receiving active therapy. She also had a history of essential arterial hypertension managed with hydrochlorothiazide and amlodipine, as well as a history of gastric ulcer. Cutaneous manifestations included Raynaud’s phenomenon, skin thickening over the forearms and lower extremities, gastroesophageal reflux, and hypo- and hyperpigmentation in facial areas. Regular outpatient follow-up during the previous three months revealed normal renal function tests.

**Figure 1:**
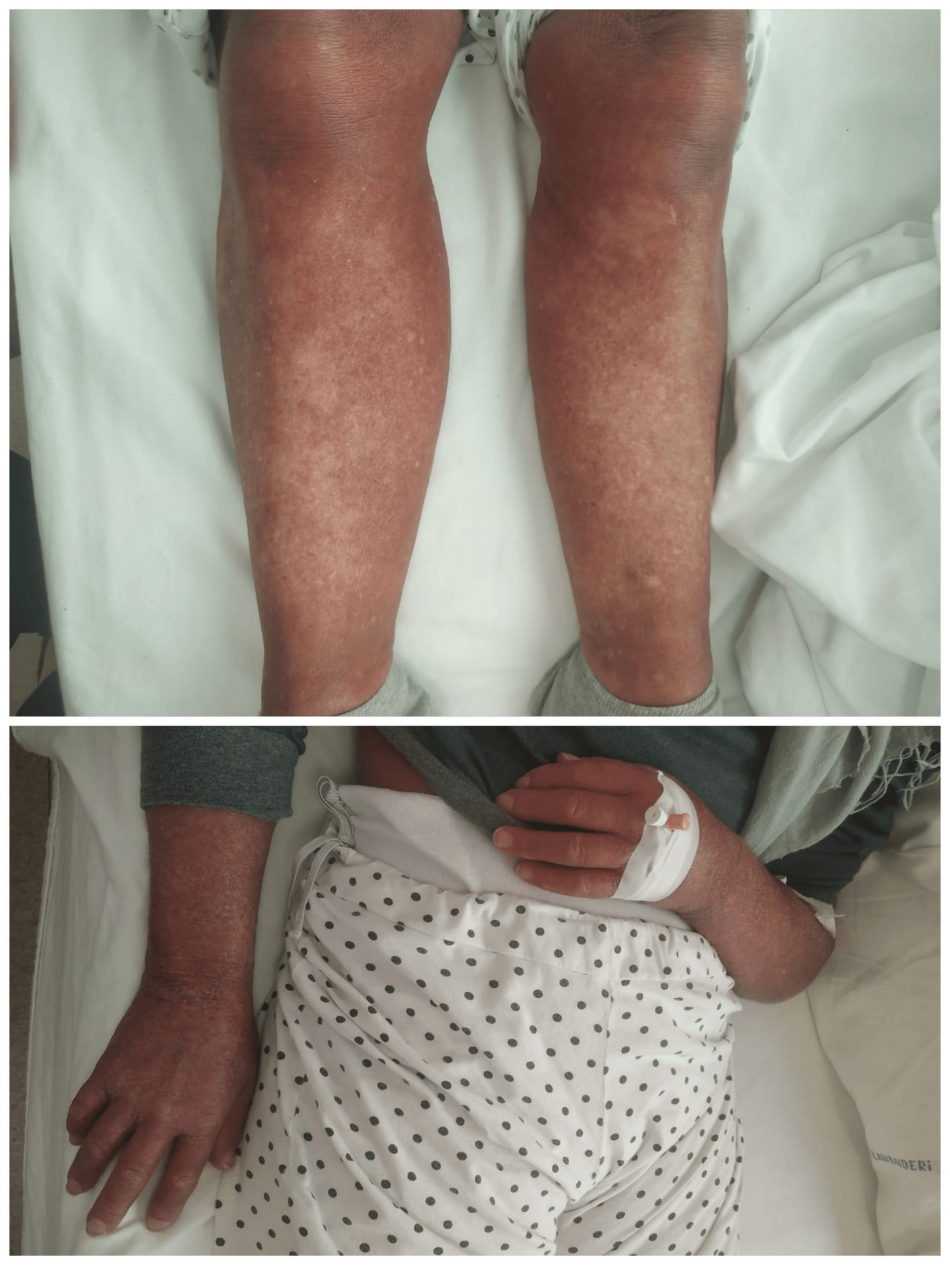
“Salt-and-pepper” skin discoloration

On admission, the patient’s blood pressure was markedly elevated at 200/100 mmHg, with a normal heart rate of 85 bpm. She was afebrile with adequate oxygen saturation (95% on room air). Diuresis was preserved and measured at approximately 150 mL/hour. Physical examination revealed no lower extremity edema but significant skin induration extending to the knees and elbows, accompanied by “salt-and-pepper” skin discoloration and sclerodactyly (Figure 1).

A comprehensive metabolic panel revealed the following findings: blood urea nitrogen (BUN): 105.1 mg/dL, serum creatinine (SCr): 3.22 mg/dL, serum sodium: 134mmol/L, serum potassium: 2.5 mmol/L, serum chloride: 92 mmol/L, serum bicarbonate: 17.7 mEq/L, serum calcium: 8.8 mg/dl, serum phosphate: 4.4 mg/dL, aspartate aminotransferase: 23 U/L, alanine aminotransferase: 20 U/L, total bilirubin: 1.42 mg/dL (with direct billirubin 0.8 mg/dL), N-terminal pro B-type natriuretic peptide (NT-proBNP): 44324.4 pg/mL, troponin I: 0.22 ng/mL, ferritin: 164 ng/mL (normal range: 30-204 ng/mL), lactate dehydrogenase (LDH): 536 IU/L, total protein: 5.7 g/dL, albuminemia: 3.4 g/dL, and alkaline phosphatase (ALP): 62 U/L. The patient's coagulation profile was within normal limits (Table 1).

**Table 1:** Metabolic panel on admission ALP: alkaline phosphatase; ALT: alanine aminotransferase; AST: aspartate aminotransferase; LDH: lactate dehydrogenase; NTproBNP: N-terminal pro B-type natriuretic peptide

Parameter	Patient values	Reference range
Urea, mg/dL	105.1	21 – 43
Creatinine, mg/dL	3.22	0.57 – 1.11
Sodium, mmol/L	134	136 – 145
Potassium, mmol/L	2.5	3.5 – 5.1
Chloride, mmol/L	92	98 – 101
Bicarbonates, mEq/L	17.7	23 – 31
Total calcium, mg/dL	8.8	8.4 – 10.2
Phosphorus, mg/dL	4.4	2.3 – 4.7
AST, U/L	23	5 – 34
ALT, U/L	20	<55
Total bilirubin, mg/dL	1.42	0.3 – 1.2
NTproBNP, pg/mL	44,324.4	<450
Troponin I, ng/mL	0.22	<0.016
LDH, IU/L	536	125 – 220
Total protein, g/dL	5.7	5.8 – 7.6
Albumin, g/dL	3.4	3.2 – 4.6
ALP, U/L	62	40 – 150
Ferritin, ng/mL	164	5 – 204
Uric acid, mg/dL	10.5	2.6 – 6

Urinalysis showed significant erythrocyturia (10-15 RBCs/HPF), clear, light-yellow urine, specific gravity of 1.006, pH of 6.0, and protein trace. As for inflammatory tests, serum procalcitonin, C-reactive protein (CRP), and erythrocyte sedimentation rate (ESR) were within normal limits. Total blood count showed a moderate anemia with hemoglobin of 8.9 g/dL (reference range: 12-16 g/dL), platelet count of 154,000/μl, and reticulocyte count of 20% (upper normal limit). Several days post-admission, haptoglobin levels were within normal range, and peripheral blood smear revealed two to three schistocytes per field. Given the severity of the patient's condition, marked by acute renal failure and a hypertensive crisis, she was promptly admitted to the Nephrology Service for specialized care and management.

Upon arrival, SRC was strongly suspected due to the patient's clinical presentation and medical history. Further rheumatological and immunological tests were performed to meticulously assess the situation and rule out other potential complications (Table 2). The presence of positive Anti-SS-A and Ro-52 antibodies indicated a possible overlap with Sjögren’s syndrome (SS). The Schirmer’s test yielded a positive result. These findings suggested an overlap between SSc and SS, which was also indicated by the Schirmer's test. Renin and aldosterone levels were assessed during hospitalization and found to be within normal ranges. Tumor markers were also evaluated, and any potential malignancy was ruled out.

**Table 2:** Immunological laboratory tests ANA: antinuclear antibody; C-ANCA: antineutrophil cytoplasmic antibody; ENA: extractable nuclear antigen antibodies; P-ANCA: antineutrophil cytoplasmic antibody; RNP: ribonucleoprotein

Laboratory test	Patient value	Normal value
ANA	1,280	<160
C-ANCA, RU/mL	1.9	<20
P-ANCA, RU/mL	1.28	<20
Rheumatoid factor-serum, IU/mL	<7.0	0 – 14.0
ENA, U/mL	185.9	<1:60
Centromeres	Negative	
Nucleosomes	Negative	
Histones	Negative	
Ribosomal P-proteins	Negative	
RNP antibody	Negative	
SS-A	Positive	
SS-B	Negative	
Scl-70	Negative	
Ro-52	Positive	
dsDNA	Negative	
C3, mg/dL	83	79 – 152
C4, mg/dL	18	16 – 38
ADAMTS13	Negative	
Hepatitis B core antibody IgM	Negative	Negative
Hepatitis B surface Ag	Negative	Negative
Hepatitis C	Negative	Negative
HIV 1 and 2 screen	Non-reactive	Non-reactive

**Figure 2:**
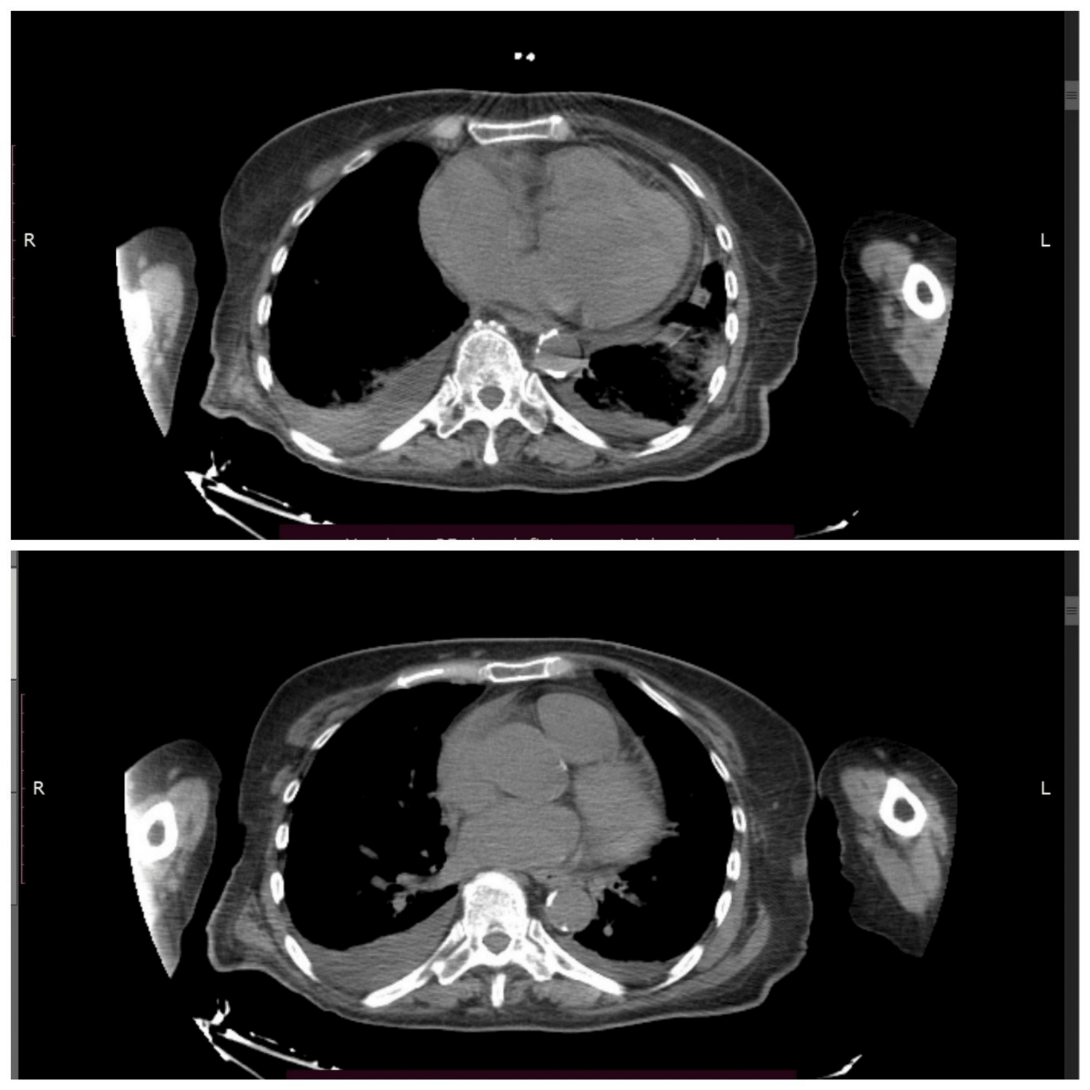
Chest CT scan showing pleural and pericardial effusion CT: computed tomography

**Figure 3:**
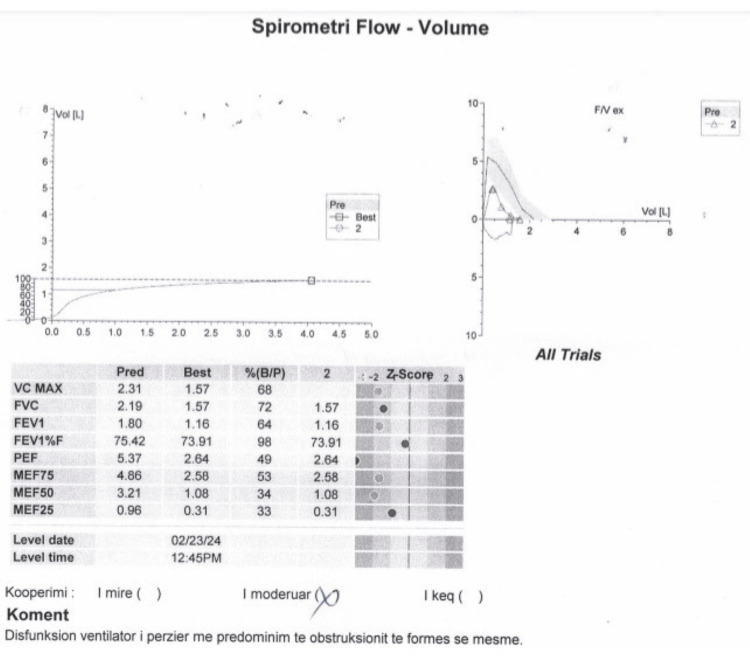
Spirometry flow-volume showing mixed ventilatory dysfunction

**Figure 4:**
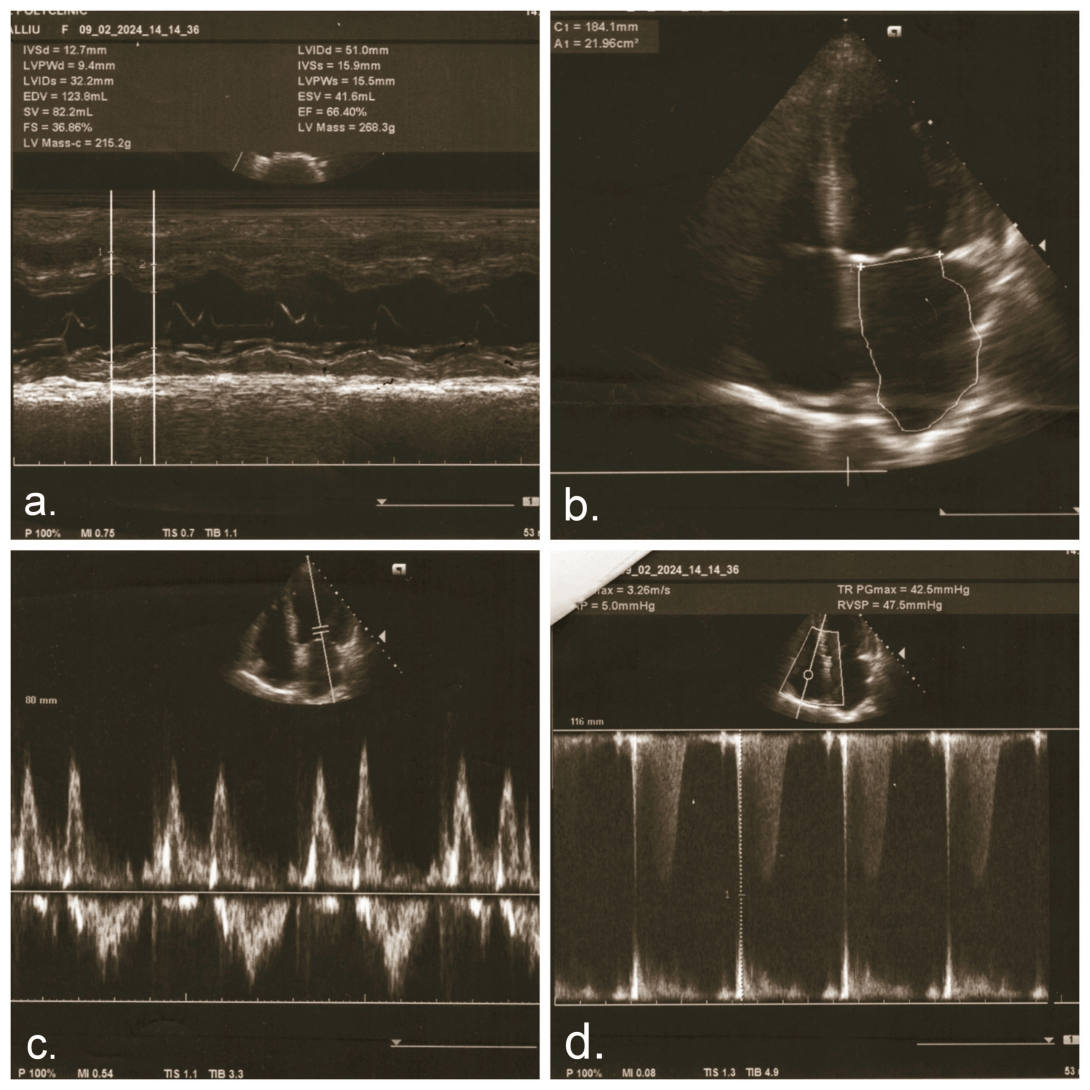
Cardiac ultrasound findings a: Normal ejection fraction. b: Left ventricular hypertrophy. c: Mitral valve regurgitation. d. Continuous wave Doppler assessment of tricuspid regurgitation

**Figure 5:**
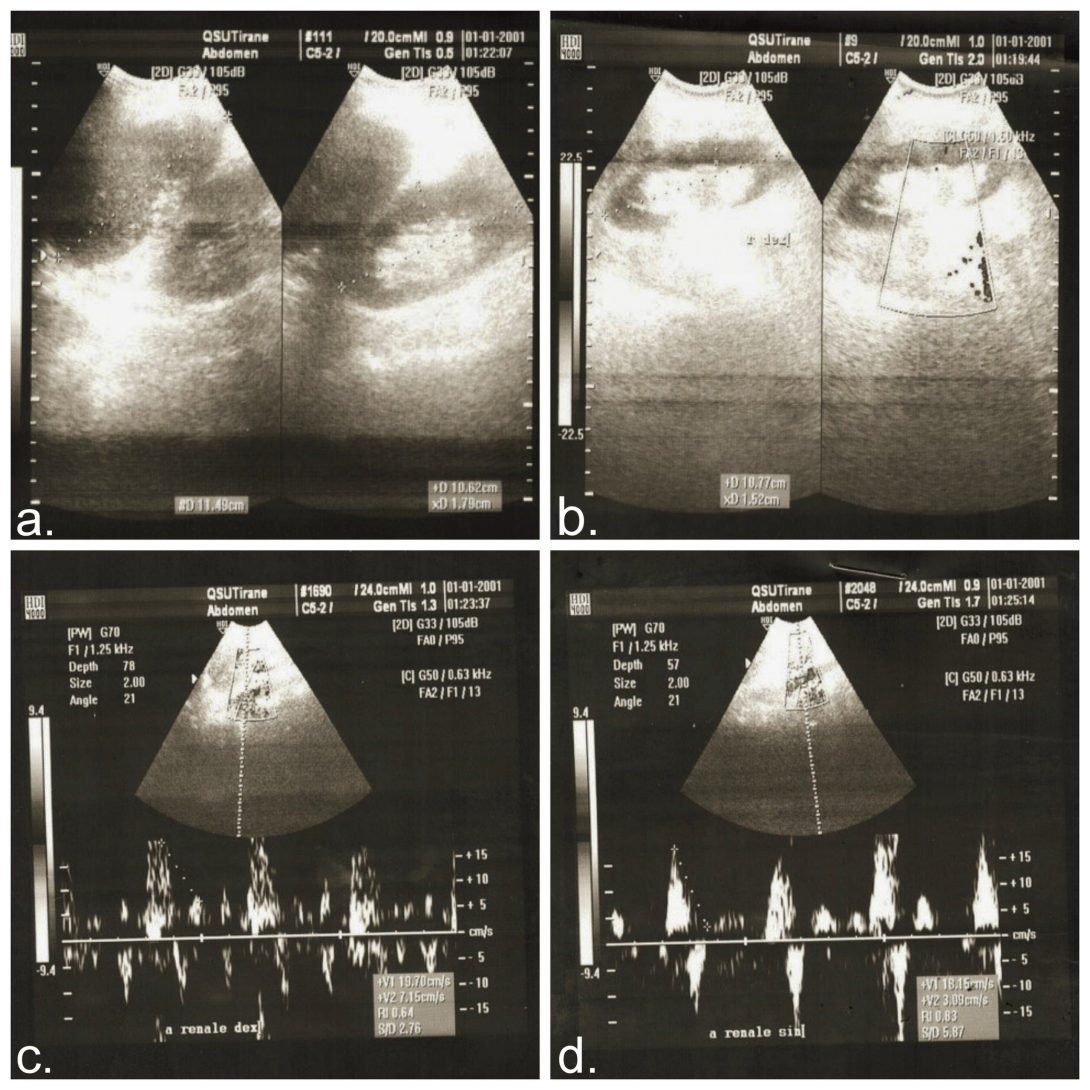
Renal ultrasound findings Left kidney (a) and right kidney (b) showing normal parenchyma. Right renal artery (c) and left renal artery (d) showing elevated peak systolic velocity, but normal renal blood flow

CT revealed bilateral pulmonary parenchymal densification and minimal fluid accumulation in the pericardial and pleural spaces (Figure 2). Spirometry indicated mixed ventilatory dysfunction, predominantly medium-sized airway obstruction (Figure 3). Cardiac ultrasonography showed left ventricular hypertrophy, aortic valve calcification with mild regurgitation, mitral valve prolapse with mild regurgitation, and pulmonary artery systolic pressures of approximately 30 mmHg (Figure 4). Renal ultrasound revealed normal renal dimensions, preserved parenchymal structure, and no evidence of renal artery stenosis or significant post-stenotic changes (Figure 5).

To confirm the diagnosis of SRC, a kidney biopsy was performed. Renal biopsy revealed 30 identified glomeruli, 10 of which exhibited global sclerosis. The remaining glomeruli showed capillary basement membrane thickening, folding, and occasional duplication, with collagenous material deposition in some areas. Arterioles demonstrated onion-skin hyperplasia with marked lumen narrowing. Immunohistochemistry revealed positivity for IgM, C3, C1q, kappa, and lambda light chains in the vessel walls.

**Figure 6:**
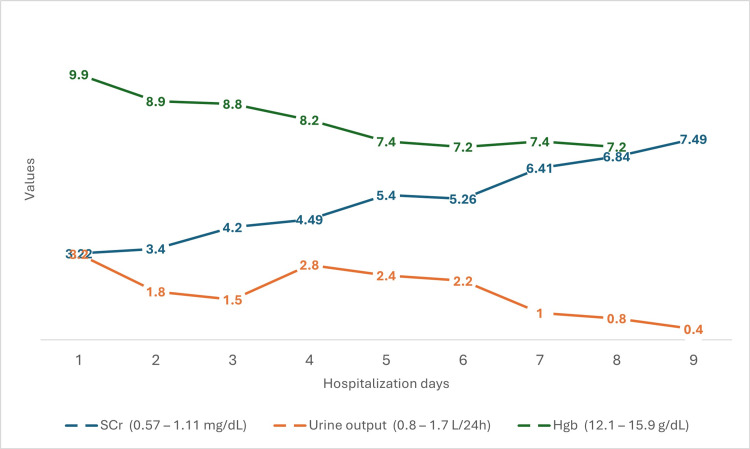
SCr, urine output, and Hgb progression during the first 10 days of hospitalization Hgb: hemoglobin; SCr: serum creatinine

Management of the hypertensive crisis proved to be a formidable challenge, necessitating a multifaceted approach demanding a complex strategy involving a wide range of antihypertensive medications. We used multiple classes of antihypertensive medications, such as calcium channel blockers (nifedipine retard), beta-blockers (bisoprolol), a weak alpha-2 receptor agonist and imidazoline I2 receptor agonist (moxonidine), and angiotensin-converting enzyme (ACE) inhibitors (initially enalapril and subsequently captopril, according to SRC treatment protocol). Despite our comprehensive and accurate medical management, hemodialysis was initiated due to deteriorating kidney function and oliguria (Figure 6).

Following stabilization, the patient was discharged with ongoing antihypertensive therapy, which was limited to captopril twice daily and outpatient hemodialysis three times weekly due to persistent renal dysfunction.

We maintained close follow-up for four months after discharge. The patient was showing signs of residual kidney function recovery, leading to a reduction of dialysis sessions to twice a week, thus giving hope for eventual discontinuation. Due to this improvement, dialysis was continued with a catheter instead of an arteriovenous fistula. However, catheter-related complications resulted in sepsis, which, in an immunosuppressed patient, proved to be fatal. Despite a period of stability reflecting the success of the therapeutic approach, the patient tragically succumbed to a systemic infectious condition, which we suspect to be a complication stemming from the hemodialysis procedure.

## Discussion

We discuss a rare presentation of SRC in the context of limited cutaneous systemic scleroderma with overlapping SS. The Diagnosis was established based on accelerated hypertension and acute renal failure, supported by characteristic renal pathology. This report highlights the critical need to consider SRC even in lcSSc cases, particularly when faced with sudden-onset hypertension and renal dysfunction. SRC is a rare yet devastating complication of SSc. Its natural history is unpredictable. While SRC is classically associated with diffuse cutaneous SSc, its occurrence in patients with limited cutaneous SSc, as seen in our patient, is less commonly reported and may be underrecognized. The combination of hypo- and hyperpigmentation, skin thickening, and Raynaud's phenomenon presents a distinctive cutaneous profile that sheds light on the uniqueness of this case. The progression of lcSSc in the absence of active therapy provides a rare opportunity to observe the natural course of the disease [1-5].

This case presented significant diagnostic challenges, with shared autoimmune features complicating differentiation among lupus, SRC, and SS. The overlap of immunological conditions made accurate diagnosis particularly difficult. The absence of anti-dsDNA, which is highly specific for lupus, further supports a diagnosis of scleroderma rather than lupus or an overlap syndrome. Additionally, the presence of characteristic skin changes, including tightening and thickening, is more suggestive of scleroderma, which can present with renal crises but lacks the typical features of lupus. On the other hand, the presence of serological markers suggestive of an overlap with SS (positive Anti-SS-A and Ro-52) adds a layer of complexity. Such overlap syndromes are relatively rare in the literature related to SRC and may influence both clinical presentation and immune-mediated vascular injury [7].

SRC pathogenesis involves complex interactions between vascular changes, activation of the renin-angiotensin-aldosterone system (RAAS), and the development of TMA. The introduction of ACE inhibitors in the 1980s marked a significant turning point, reducing the one-year mortality rate to 24%. These medications, such as captopril and enalapril, are the cornerstone of therapy for SRC. They work by inhibiting the conversion of angiotensin I to angiotensin II, thereby lowering blood pressure and improving renal function. Early initiation of ACE inhibitors is crucial for stabilizing blood pressure and improving renal function, even in patients with severely impaired kidney function. This has changed the outlook for patients with SRC, shifting the focus from managing a deadly condition to one in which many patients can recover significantly and improve their quality of life [7-8].

Several risk factors are associated with the development of SRC, like recent high-dose corticosteroid use, high skin thickening scores, and the presence of specific autoantibodies. The presence of specific autoantibodies, particularly anti-RNA polymerase III (anti-RNAP III) antibodies, plays a pivotal role and is highly associated with an increased risk of SRC. Several studies have found that anti-RNAP III antibodies are detectable in up to 52% of patients with SRC. These antibodies actively cause immune-mediated damage to blood vessels, which leads to severe hypertension and the production of dangerous blood clots. Although further tests found no anti-RNA polymerase III antibodies in our patient, and she had no corticosteroid exposure, her illness demonstrated the multifaceted nature of SRC. This complexity led to rapid kidney failure and severe anemia due to the destruction of red blood cells [7-11].

Renal biopsy is a critical diagnostic tool in SRC, providing detailed insights into the underlying pathology and guiding treatment decisions. This is particularly important in cases where the clinical presentation is atypical or overlaps with other conditions, as seen in our case with Sjögren's overlapping syndromes, adding further complexity to the diagnostic framework. Such overlap syndromes are relatively rare in the SRC-related literature and may influence clinical presentation and immune-mediated vascular injury. Based on the renal biopsy report, the findings were much more indicative of SRC due to the highly characteristic and severe arteriolar "onion-skin hyperplasia” and the presence of TMA patterns consistent with the observations in this case. The absence of specific glomerular immune complex deposition (e.g., "full house" pattern in glomeruli) strongly supports SRC over classic lupus nephritis (LN). The IHC specifically states positivity in the vessel walls, not within the glomerular immune deposits, which are the hallmark of LN. However, immunohistochemical findings, including IgM and complement deposition, suggest an added dimension of immune-mediated vascular injury, underscoring the awkward interplay between pathology and the immune system. This information is crucial for tailoring the treatment plan and predicting the response to therapy [9,11-13].

Standard treatment protocols for SRC typically focus on the use of ACE inhibitors, which have been a cornerstone therapy for decades. However, the clinical picture can be significantly complicated by the presence of other autoimmune features or comorbid conditions. This tailored approach ensures that treatment is optimized for each individual, addressing the complexity of their condition and improving outcomes. Nonetheless, given its potentially fulminant nature, certain cases may be refractory to conventional treatment, emphasizing the need for earlier recognition and possibly more individualized therapeutic approaches [14].

Despite advancements, the progression to end-stage renal disease (ESRD) requiring hemodialysis is not uncommon in this patient population, particularly in cases with delayed intervention [15]. The mortality remains high, often due to complications such as infections, as highlighted by the fatal outcome in our patient. Infection risk is exacerbated in patients undergoing hemodialysis due to frequent vascular access and compromised immunity. Multidisciplinary care, involving rheumatologists, nephrologists, and other specialists, and close monitoring are essential to improving outcomes [4,16-17].

## Conclusions

This report underscores the critical need for early recognition of SRC, as rapid diagnosis and aggressive treatment can prevent severe complications. Managing SRC is highly challenging due to disease progression risks and treatment-related complications, further complicated by overlapping features with SS, which strengthens the presence of shared autoimmune mechanisms, making diagnosis and intervention more complex. However, early identification, proactive management of hypertensive crises and renal impairment, and continuous monitoring can significantly improve patient outcomes and quality of life. Ultimately, this report highlights the importance of heightened clinical suspicion for SRC in limited cutaneous SSc, particularly in autoimmune overlap syndromes, while stressing the need for refined therapeutic approaches in preventing poor outcomes despite early intervention.
